# Cannabidiol (CBD) content in vaporized cannabis does not prevent tetrahydrocannabinol (THC)-induced impairment of driving and cognition

**DOI:** 10.1007/s00213-019-05246-8

**Published:** 2019-05-01

**Authors:** Thomas R. Arkell, Nicholas Lintzeris, Richard C. Kevin, Johannes G. Ramaekers, Ryan Vandrey, Christopher Irwin, Paul S. Haber, Iain S. McGregor

**Affiliations:** 1 Lambert Initiative for Cannabinoid Therapeutics, The University of Sydney, Sydney, New South Wales Australia; 2 Brain and Mind Centre, The University of Sydney, Sydney, New South Wales Australia; 3 Faculty of Medicine, Central Clinical School, The University of Sydney, Sydney, New South Wales Australia; 4 The Langton Centre, Drug and Alcohol Services, South East Sydney Local Health District, NSW Health, Sydney, New South Wales Australia; 5 Faculty of Science, School of Psychology, The University of Sydney, Sydney, New South Wales Australia; 6 Faculty of Psychology and Neuroscience, Maastricht University, Maastricht, The Netherlands; 7 Johns Hopkins University School of Medicine, Baltimore, MD USA; 8 School of Allied Health Sciences, Griffith University, Gold Coast, Australia; 9 Drug Health Services, Royal Prince Alfred Hospital, Camperdown, New South Wales Australia

**Keywords:** Cannabis, Tetrahydrocannabinol, Cannabidiol, THC, CBD, Driving, Cognition

## Abstract

**Background:**

The main psychoactive component of cannabis, delta-9-tetrahydrocannabinol (THC), can impair driving performance. Cannabidiol (CBD), a non-intoxicating cannabis component, is thought to mitigate certain adverse effects of THC. It is possible then that cannabis containing equivalent CBD and THC will differentially affect driving and cognition relative to THC-dominant cannabis.

**Aims:**

The present study investigated and compared the effects of THC-dominant and THC/CBD equivalent cannabis on simulated driving and cognitive performance.

**Methods:**

In a randomized, double-blind, within-subjects crossover design, healthy volunteers (*n* = 14) with a history of light cannabis use attended three outpatient experimental test sessions in which simulated driving and cognitive performance were assessed at two timepoints (20–60 min and 200–240 min) following vaporization of 125 mg THC-dominant (11% THC; < 1% CBD), THC/CBD equivalent (11% THC, 11% CBD), or placebo (< 1% THC/CBD) cannabis.

**Results/outcomes:**

Both active cannabis types increased lane weaving during a car-following task but had little effect on other driving performance measures. Active cannabis types impaired performance on the Digit Symbol Substitution Task (DSST), Divided Attention Task (DAT) and Paced Auditory Serial Addition Task (PASAT) with impairment on the latter two tasks worse with THC/CBD equivalent cannabis. Subjective drug effects (e.g., “stoned”) and confidence in driving ability did not vary with CBD content. Peak plasma THC concentrations were higher following THC/CBD equivalent cannabis relative to THC-dominant cannabis, suggesting a possible pharmacokinetic interaction.

**Conclusions/interpretation:**

Cannabis containing equivalent concentrations of CBD and THC appears no less impairing than THC-dominant cannabis, and in some circumstances, CBD may actually exacerbate THC-induced impairment.

**Electronic supplementary material:**

The online version of this article (10.1007/s00213-019-05246-8) contains supplementary material, which is available to authorized users.

## Introduction

With the growing worldwide trend towards the decriminalization of recreational and medicinal cannabis use, there has been a renewed focus on the risks associated with driving under the influence of cannabis (Ramaekers 2018; Capler et al. 2017). Epidemiological studies suggest that cannabis intoxication produces a modest increase in the risk of being involved in a crash (Rogeberg and Elvik 2017; Rogeberg and Elvik 2016) although not the risk of being seriously injured or killed (Schulze et al. 2012). On-the-road and laboratory studies of driving performance consistently show that cannabis tends to impair driving-related skills and cognitive functions in a dose-dependent manner (Veldstra et al. 2015; Bosker et al. 2012; Lamers and Ramaekers 2001; Downey et al. 2013; Hartman et al. 2015).

These experimental studies of driving performance have typically involved administration of smoked cannabis containing Δ^9^-tetrahydrocannabinol (THC), or pharmaceutical THC given in a capsule form (e.g., dronabinol) (Veldstra et al. 2015; Bosker et al. 2012; Lamers and Ramaekers 2001; Downey et al. 2013; Hartman et al. 2015; Papafotiou et al. 2005). However, as the therapeutic effects of the non-intoxicating cannabinoid cannabidiol (CBD) become more apparent (Zhornitsky and Potvin 2012; Cuba et al. 2017; Mannucci et al. 2017), it is likely that community use of medicinal cannabis products containing both THC and CBD will become increasingly common.

There is speculation, and some evidence, that CBD may minimize some of the negative side effects associated with THC and enhance therapeutic efficacy (Hindocha et al. 2015; Martin-Santos et al. 2012; Bhattacharyya et al. 2010; Russo and Guy 2006). CBD has sometimes been found to lessen the “euphoria” associated with cannabis intoxication (Dalton et al. 1976), and attenuate THC-induced attentional bias towards food and drug-related stimuli (Morgan et al. 2010b). CBD can lessen THC-induced impairment of facial emotion recognition (Hindocha et al. 2015) and improve such recognition when administered alone. Naturalistic studies suggest that cannabis users consuming higher CBD products experience fewer psychotic experiences (Schubart et al. 2011), fewer positive schizophrenia-like symptoms (Morgan and Curran 2008), and less memory-impairment (Morgan et al. 2010a).

Other studies, however, have failed to observe modulatory effects of CBD on THC subjective drug effects (e.g., being “stoned”) when the CBD is smoked (Morgan et al. 2010a; Ilan et al. 2005), vaporized (Hindocha et al. 2015), or administered orally (Roser et al. 2008; Juckel et al. 2007; Hollister and Gillespie 1975; Haney et al. 2016). In one study, pre-treatment with CBD (up to 800 mg oral) did not alter the physiological or subjective effects of smoked THC-dominant cannabis (Haney et al. 2016). Preclinical studies indicate that CBD can even sometimes potentiate some of the behavioral and cognitive effects of THC, possibly by way of a pharmacokinetic interaction whereby CBD potentiates blood THC levels (Boggs et al. 2018; Klein et al. 2011).

The above inconsistencies highlight the need for human studies that systematically compare the effects of different cannabis chemovars (“strains”) containing varying amounts of THC and CBD. In jurisdictions where medicinal and/or recreational cannabis is widely available, users often have a choice of hundreds of such types of cannabis, which vary with respect to THC and CBD content. This study therefore sought to compare the subjective, cognitive, and driving-related effects of vaporized THC-dominant (11% THC, < 1% CBD [hereafter “THC”]), THC/CBD-equivalent (11% THC, 11% CBD [hereafter “THC/CBD”]) and placebo (< 1% THC; <1% CBD) cannabis.

## Methods

### Study design and procedures

This double-blind, within-subjects crossover study included three experimental sessions that were scheduled at least 7 days apart to avoid carryover effects. Participants received the three treatments (one per session) in a randomized and counterbalanced order. The randomization schedule was created by an independent researcher, and only the study pharmacist had access to it. Between 1 and 4 weeks prior to the first session, participants attended an orientation session in which they practiced the driving simulation and cognitive tasks. Practice was continued until participants demonstrated competence in each task. Participants were instructed to abstain from illicit drugs for the duration of the study (i.e., from the time of study enrollment until the final session) and from alcohol on the night before research sessions, and to maintain any use of regular medications. Participants were also instructed to consume no more than their regular caffeine intake on the morning of research sessions.

### Participants

Participants were healthy adults with a history of infrequent cannabis use. Inclusion criteria were aged 18–65 years, self-reported cannabis consumption ≤ 2 times/week in the previous 3 months and ≥ 10 lifetime exposures, and possession of and minimum 1-year driving on an unrestricted Australian license (i.e., > 4 years total driving experience). Exclusion criteria included current mood disorder, lifetime major psychiatric illness, history of clinically significant adverse response to previous cannabis exposure, any moderate or severe substance use disorder as assessed by an addiction medicine specialist, pregnant/nursing, interest in treatment to reduce cannabis use, current use of medications known to affect driving, active hypertension, cardiovascular disease, or chronic pulmonary disease. Volunteers were recruited through online advertisement, social media (e.g., Facebook), and word of mouth. After an initial phone screen, participants meeting inclusion/exclusion criteria were invited to attend a medical screen which involved a detailed medical and psychiatric evaluation. All participants gave written informed consent prior to study enrollment. All procedures were approved by the Sydney Local Health District (RPAH Zone) Human Research Ethics Committee and were in accordance with the Declaration of Helsinki. The trial was listed on the Australia New Zealand Clinical Trials Registry (No. 12616000414415).

### Experimental sessions

**Fig. 1:**
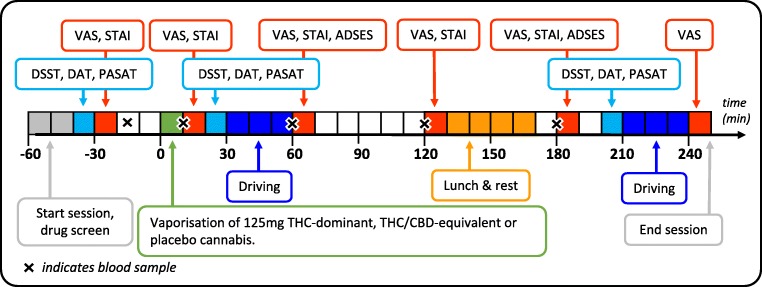
Order of events during experimental sessions. VAS visual analog scale, STAI State Trait Anxiety Inventory, DSST Digit Symbol Substitution Task, DAT Divided Attention Task, PASAT Paced Auditory Serial Addition Task, ADSES Adelaide Driving Self-Efficacy Scale

The order of events during research sessions is presented in Fig. 1. Participants arrived at the clinical research unit at 09:00 on the morning of research sessions. Nil breath alcohol concentration (BrAC) was confirmed via breathalyzer (Alcotest 5510, Draeger, Lübeck, Germany), and oral fluid was screened (DrugWipe 5s, Securetec, Neubiberg, Germany) to rule out recent drug use. Participants testing positive for any drug (cannabis, cocaine, opiates, or amphetamines/MDMA/methamphetamines) were sent home and the session was rescheduled. Participants completed a baseline questionnaire at the start of each session which asked about recent use of drugs, alcohol and caffeine, adverse events since the previous session, and perceived sleep quality during the previous night. Baseline cognitive task performance and subjective drug effects were assessed 30 min prior to dosing.

Participants inhaled 125 mg THC (11% THC, < 1% CBD), THC/CBD (11% THC, 11% CBD), or placebo (< 1% THC, < 1% CBD) cannabis (Tilray, BC, Canada) via vaporization at 200 °C (Mighty Medic, Storz & Bickel, Tuttlingen, Germany) over 5 min according to a standardized procedure (inhale 3 s, hold 3 s, exhale, and rest 30 s). If vapor was still visible in exhaled breath at 5 min, then this procedure was continued until vapor was no longer visible.

### Blood collection and plasma cannabinoid levels

Blood was collected via indwelling peripheral venous catheter into purple-top (EDTA) Vacutainer® tubes (Becton, Dickinson and Company, Franklin Lakes, NJ) 20 min prior to and 10-, 60-, 120-, and 180-min post-inhalation. The blood was centrifuged at 1228×*g* for 10 min and the supernatant plasma was decanted and stored in 3.6-mL Nunc® cryotubes (Thomas Scientific, Swedesboro, NJ) at − 80 °C until analysis. Plasma was subsequently thawed for analysis via liquid chromatography-tandem mass spectrometry (LC-MS/MS) according to previously published methods (Kevin et al. 2017; Schwope et al. 2011). Duplicate 200-μL plasma aliquots were fortified with an internal standard mixture containing *d*_3_-THC, *d*_3_-THC-COOH, and *d*_3_-11-OH-THC. Duplicate calibrator samples were prepared with cannabinoid-free plasma (obtained from the Red Cross), spiked with appropriate amounts of a standard mixture of THC, 11-OH-THC, THC-COOH, CBD, and internal standards to generate a standard curve and quality control samples for each analyte. All samples were diluted 1:1 in 0.1% formic acid in water, and analytes were extracted using 400 μL capacity ISOLUTE SLE+ supported liquid extraction columns (Biotage, Sydney, Australia). The analytes were eluted with 500 μL dichloromethane, 300 μL ethyl acetate, and 1.2 mL methyl *tert-*butyl ether. The eluate was evaporated under a gentle stream of nitrogen without heating, and analytes were reconstituted in 100 μL of 40:60 acetonitrile and 0.1% formic acid in water, transferred to 2-mL autosampler vials fitted with 100-μL glass inserts, and placed in the LC-MS/MS autosampler held at 4 °C. Chromatographic separation was achieved using an Eclipse XDB-C18 column (50 mm × 2.1 mm i.d., particle size 3.5 μm; Agilent Technologies, Singapore) using gradient elution with mobile phases 0.1% formic acid in water and acetonitrile, at a flow rate of 0.3 mL/min. This was coupled to a Shimadzu LCMS-8030 mass spectrometer for analyte identification and quantification.

### Driving simulator

The custom-built driving simulator (Hyperdrive, Adelaide, Australia) consisted of a fixed-base equipped with original vehicle controls (steering wheel, indicators, seat, safety belt), hi-resolution Fanatec® pedals, and a servo motor wheel base (Endor AG, Landshut, Germany) linked to four networked computers running SCANeR™ Studio simulation engine (v1.6, OKTAL, Paris, France). Visual images were displayed on three 32-in. LCD monitors using three channels set to provide a 100^°^ field of view. A digital dashboard displayed speed, rpm, and status of vehicular control systems (e.g., traction control). A complete rear visual scene was displayed on three separate channels (rear vision mirror, left, and right-side mirrors). Graphics refreshed at a rate of 60 Hz, and data were sampled at a rate of 20 Hz. Surround sound provided auditory feedback, and force feedback steering provided haptic feedback. Data collected by the simulator’s software program included measures of lateral control (lateral position, steering wheel angle), longitudinal control (speed, acceleration), and interaction with other vehicles.

### Driving scenarios

The driving simulation started with a 5-min highway car-following task in which participants were required to follow and maintain a constant distance (headway) to a lead vehicle that would accelerate or decelerate every 30 s in a sinusoidal manner (between 90 and 110 km/h). The task occurred on a two-lane, dual-carriageway highway in steady highway traffic. Outcome measures included standard deviation of lateral position (SDLP; a measure of lane weaving (Verster and Roth 2011)), mean headway (i.e., distance to the lead vehicle), and standard deviation of headway.

The remainder of the task (25 min; “secondary driving task”) consisted of highway and rural segments. Participants were instructed to follow the spoken GPS directions and drive as they normally would. Highway segments involved a two-lane, dual-carriageway road with posted 110 km/h speed limits, rural segments involved winding single-lane roads with various posted speed signs (60–100 km/h), and intersections with and without signal-controls. Outcome measures included SDLP, mean speed (MSP), and standard deviation of speed (SDSP). Throughout the task, there were various hazards (e.g., roadworks, aggressive drivers), cyclists, pedestrians, and traffic in variable density. To minimize familiarity, the appearance (i.e., make, model and color) of other vehicles was generated randomly for each drive. The time of day for each drive was set to match the actual time of testing. Tests of simulated driving began 30 min (T1) and 210 min (T2) after dosing.

### Cognitive tasks

Cognitive/psychomotor performance was assessed using three computerized tasks which are known to be sensitive to the impairing effects of THC (Vandrey et al. 2017). These included the Digit Symbol Substitution Task (DSST; (Mcleod et al. 1982)), Divided Attention Task (DAT; (Kleykamp et al. 2010)), and Paced Auditory Serial Addition Task (PASAT; (Herrmann et al. 2015)). Performance assessments were completed in this order at baseline and 20 min (T1) and 200 min (T2) after dosing.

In the DSST, participants were presented with a series of geometric patterns labeled from 1 to 9, each consisting of an array of filled and blank squares in a 3 × 3 grid. When a number appeared in the middle of the screen, participants were instructed to replicate the pattern corresponding to that array using the numeric keypad of a computer keyboard. Participants had 90 s to replicate as many patterns as possible. Outcome measures included number of patterns correct and accuracy (number of patterns correct/number of patterns attempted).

In the DAT, participants were required to track a horizontally moving stimulus on the screen using their mouse while simultaneously responding to peripheral visual stimuli by clicking the left mouse button whenever a number in any corner of the screen matched a target number presented at the bottom of the screen. Outcome measures included mean distance of the cursor from the target (tracking error), the number of target numbers correctly identified (/24), and response time.

In the PASAT, participants watched single digits appear on the screen and were instructed to sum each new digit with the preceding one. Participants responded by clicking on the correct answer from a list of numbers (1–10) presented on the screen. Outcome measures included response time on correct trials and the total number of correct trials (/90).

### Subjective drug effects

Subjective drug effects were assessed at baseline and 15, 60, 120, 180, and 240 min after dosing using a series of Visual Analog Scales (VAS). Participants rated on a 100- mm line their responses to the statements: “Strength of drug effect”, “Liking of drug effect”, “Stoned,” “Sedated,” “Anxious,” and “Confident to drive”. All scales were unipolar except for “Liking of drug effect” which was bipolar (dislike very much – like very much). The State-Trait Anxiety Inventory (Y-1 – state form only) (Spielberger 1983) was also administered at baseline and 15, 60, 120 and 180 min after dosing. Self-reported driving ability was further assessed by the Adelaide Driving Self-Efficacy Scale (George et al. 2007) (which provides a score from 0 to 120) at baseline and at 60 and 180 min after dosing.

### Statistical analysis

Sample size was determined by a power calculation based on the effect size (ηp^2^ = 0.14) associated with the main effect of Dronabinol on simulated driving performance reported in a previous study (Veldstra et al. 2015). Data from the driving simulator tasks were reviewed and cleaned to remove recognizable artifacts (e.g., increased SDLP while overtaking other vehicles). All data were analyzed in SPSS v24 (IBM Corp, Armonk, NY) with Linear Mixed Models (LMMs). The restricted maximum likelihood method was selected, and a first-order autoregressive (AR1) covariance structure was specified for repeated factors as it provided the lowest Akaike information criterion (AICC) model fit values. Fixed factors included treatment (3 levels), time (2, 3, and 5 levels for driving, cognitive and pharmacokinetic/subjective drug effects data, respectively), session (3 levels), and treatment by time. For data which included baseline assessment (i.e., cognitive, pharmacokinetic, and subjective drug effect data), baseline scores were included in the model as a covariate. In each model, planned Bonferroni pairwise comparisons were used to compare treatment means at each level of time. For additional pharmacokinetic data (e.g., area under the curve), non-parametric Wilcoxon signed-rank tests were used to assess differences between treatments. The statistical significance level was set at *p* < 0.05.

## Results

### Participants

Table 1 presents the characteristics of the 14 healthy adults (11 males, 21–38 years old) who completed all three test sessions between December 2017 and April 2018. Three additional participants began, but did not complete, the study: one withdrew after the first session, another was discharged for protocol non-compliance, and another was discharged prior to drug administration following multiple elevated baseline blood pressure and heart rate readings. None of the 14 participants reported regular use of any medications, and all participants provided negative oral fluid drug tests on the morning of each test session. Blinding was assessed at the end of the trial by asking participants which cannabis type they thought they received in each of the three sessions. All 14 participants correctly identified the placebo session, and it was commonly reported that less vapor was produced by the placebo cannabis.

**Table 1:** Characteristics of participants

Number of participants	14
Sex (male/female)	11/3
Race (White/Asian)	13/1
Age (years)	27.5 (4.5)
BMI (kg/m^2^)	25.5 (4.9)
Alcohol intake frequency (no. of days per month)	7.1 (5.3)
Age of first cannabis use (years)	15.9 (2.6)
Days used cannabis in last 28 days (no.)	4.5 (4.8)
Days used cannabis use in last 3 months (no.)	11.2 (8)
Years of driving experience (no.)	9.6 (4.1)
Total days driven in last 28 days (no.)	11.9 (10.6)
Typical wait before driving after consuming cannabis (h)	5.9 (7)

Data are shown as means (SD) or as frequency. *BMI* body mass index

### Subjective measures

**Fig. 2:**
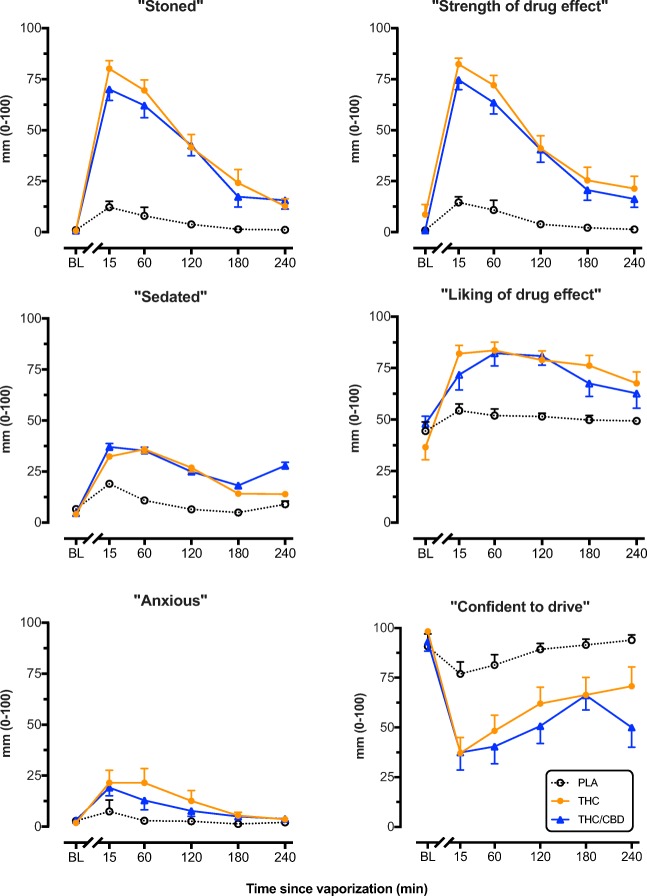
Mean (SEM) participant ratings of “Stoned”, “Strength of drug effect”, “Sedated”, “Liking of drug effect”, “Anxious” and “Confident to drive” assessed using 1-100mm visual analog scales after vaporization of placebo, THC-dominant, and THC/CBD-equivalent cannabis. All scales were unipolar except for “Liking of drug effect”. *BL* baseline

As shown in Fig. 2, there was a significant main effect of treatment on “Stoned” [*F*(2, 43.27) = 64.25, *p* < 0.001], “Strength of drug effect [F(2,45.81) = 70.24, *p* < .001], “Sedated” [*F*(2, 59.18) = 15.25, *p* < .001] and “Liking of drug effect” [*F*(2, 49.43) = 17.53, *p* < 0.001]. Subjective ratings did not differ between the THC and THC/CBD conditions at any point in time, however only THC/CBD increased ratings of “Sedated” when compared with placebo at 240 min (*p* = 0.008). Treatment also significantly affected ratings of “Anxious” [*F*(2, 44.52) = 5.40, *p* = 0.008] (Fig. 2) and STAI scores *[F*(2,51.08) = 7.05, *p* = 0.002]. On both measures, both THC and THC/CBD produced a modest but significant increase in ratings of “Anxious” at 15 min when compared with placebo, but only THC did so at 1 h. Finally, treatment significantly affected ratings of “Confident to drive” [F(2,53.52) = 27.68, *p* < .001] (Fig. 2) and Adelaide Driving Self-Efficacy Scale scores [F(2,108.417) = 20.41, *p* < .001] (Fig. S1) such that both THC and THC/CBD significantly decreased scores on both measures when compared with placebo up to 240 min after vaporizing.

#### Driving performance

Mean (SD) values of driving outcome measures are presented in Table 2.

**Table 2:** Driving performance [means (SD)] at T1 and T2 (30 min and 210 min) after vaporization of placebo, THC-dominant, and THC/CBD-equivalent cannabis

	T1			T2		
	Placebo	THC	THC/CBD	Placebo	THC	THC/CBD
Car-following task						
SDLP (cm)	19.29 (6.47)	23.21 (4.77)^*^	23.50 (7.36)^*^	19.00 (5.11)	22.86 (6.00)^*^	24.21 (6.39)^**^
Headway (m)	75.38 (23.32)	85.28 (30.48)	86.01 (41.48)	71.82 (23.81)	82.91 (27.04)	90.13 (36.59)
SD headway (m)	29.10 (11.92)	30.82 (12.97)	40.59 (25.97)	28.22 (13.50)	34.33 (14.67)	42.79 (24.75)^*^
Secondary driving task						
SDLP (cm)	27.07 (5.41)	28.43 (5.52)	28.5 (6.62)	28.43 (6.31)	28.36 (5.79)	28.71 (6.34)
Speed (km/h)	82.16 (4.02)	81.47 (3.17)	82.72 (3.48)	84.21 (3.11)	83.72 (3.56)	83.46 (3.41)
SDSP (km/h)	26.96 (3.46)	27.28 (2.19)	25.78 (1.88)	26.91 (2.34)	26.30 (2.17)	26.45 (1.57)

*SDLP* standard deviation of lateral position, *SD distance* standard deviation of distance, *SDSP* standard deviation of speed. Bonferroni post hoc tests were used to compare treatment means at each level of time. ^*^Indicates significantly different from placebo (*p* < 0.05). ^**^Indicates significantly different from placebo (*p* < 0.01)

##### Car-following task

In the car-following section of the driving task, there was a significant main effect of treatment on SDLP [F(2,31.43) = 11.44, *p* < 0.001] but no treatment by time interaction. When compared with placebo, THC increased SDLP by 3.91 cm (*p* = 0.019) and 3.84 cm (*p* = 0.041) at T1 and T2, respectively, while THC/CBD increased SDLP by 4.28 cm (*p* = 0.036) and 5.28 cm (*p* = 0.003). Mean headway (i.e. distance to the lead vehicle) did not differ between conditions [F(2,30.17) = 2.73, *p* = 0.081], however standard deviation of headway did [F(2, 27.743) = 4.96, *p* = 0.014]. When compared with placebo, the THC/CBD condition had greater standard deviation of headway at T2 by 15.33 m (*p* = 0.044).

##### Secondary driving task

In the secondary driving task, treatment did not significantly affect lateral or longitudinal vehicular control parameters including SDLP, MSP and SDSP. Separate analyses of SDLP over different segments (i.e. highway and rural) of the drive indicated that cannabis did not significantly impair driving at any point during the 25 min task.

#### Cognitive task performance

Mean (SD) values of cognitive task performance measures are presented in Table 3.

**Table 3:** Cognitive task performance [means (SD)] at baseline, T1, and T2 (20 min and 200 min) after vaporization of placebo, THC-dominant, and THC/CBD-equivalent cannabis

	Baseline			T1			T2		
	Placebo	THC	THC/CBD	Placebo	THC	THC/CBD	Placebo	THC	THC/CBD
DSST									
Number correct	47.21 (7.06)	47.86 (7.93)	47.14 (8.66)	49.07 (6.67)	44.79 (8.29) ^*^	45.36 (7.04)	50.57 (6.81)	50.5 (6.02)	50.14 (5.49)
Accuracy (%)	94.99 (4)	95.25 (4.56)	95.01 (6.42)	95.91 (3.64)	92.07 (6.32)	93.08 (6.85)	97.31 (3.73)	95.35 (3.61)	96.84 (4.72)
DAT									
Tracking error (pixels)	10.94 (1.98)	11.45 (3.34)	11.58 (3.13)	13.19 (3.54)	14.17 (3.75)	16.44 (7.29) ^*,#^	14.11 (3.02)	14.37 (3.34)	14.76 (2.91)
Response time (ms)	905.89 (179.36)	929.65 (334.47)	911.01 (159.45)	972.61 (248.56)	937.74 (258.91)	1026.79 (261.53)	961.15 (204.19)	1027.64 (307.62)	1029.95 (245.91)
Number correct	23 (1.02)	23 (1.22)	23.14 (1.01)	23.07 (1.14)	22.64 (2.37)	22.93 (1.07)	22.93 (2.02)	22.14 (2.8)	23.36 (1.08)
PASAT									
Number correct	76.36 (9.2)	78.57 (11.41)	75.43 (12.71)	81.86 (7.8)	77.14 (14.04)	71.5 (16.58) ^**^	79.5 (9.2)	78.29 (14.38)	76.86 (12.32)
Response time (ms)	1308.2 (125.69)	1306.83 (128.73)	1331.35 (129.41)	1291.47 (79.84)	1429.14 (153.48) ^**^	1451.13 (174.06) ^**^	1328.64 (108)	1324.42 (142.38)	1347.6 (101.38)

*DSST* Digit Symbol Substitution Task, *DAT* Divided Attention Task, *PASAT* Paced Auditory Serial Addition Task

^*^Indicates significantly different from placebo (*p* < 0.05). ^**^Indicates significantly different from placebo (*p* < 0.01). ^#^Indicates significantly different from THC (*p <* 0.05)

##### DSST

**Fig. 3:**
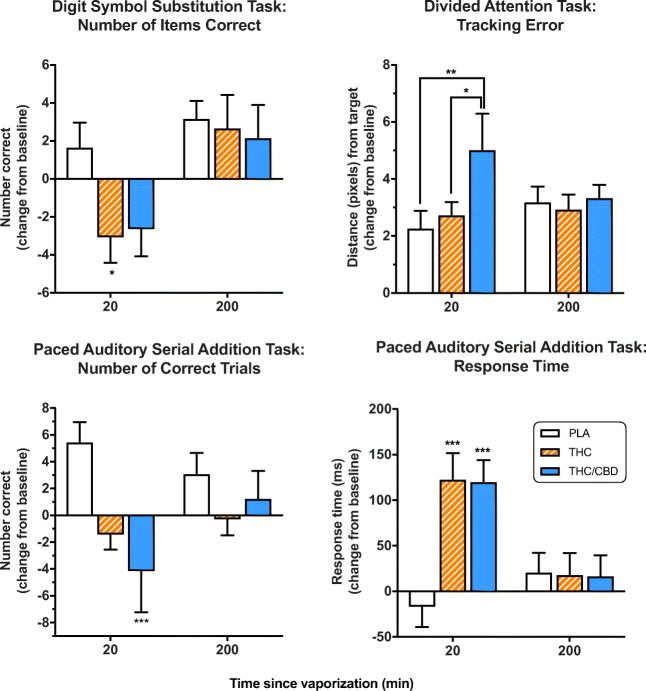
Mean (SEM) performance on the Digit Symbol Substitution Task (DSST), Divided Attention Task (DAT), and Paced Auditory Serial Addition Task (PASAT) after vaporization of placebo, THC-dominant, and THC/CBD-equivalent cannabis. *p < 0.05, **p < 0.01, ***p < 0.001, Bonferroni post-hoc. *BL* baseline

In the DSST, there was a trend towards a main effect of treatment on accuracy [*F*(2,50.52) = 2.80, *p* = 0.07] and number of items correct [*F*(2,49) = 2.55, *p* = 0.09]. Planned Bonferroni comparisons showed that participants exhibited fewer correct items at T1 in the THC condition when compared with placebo (*p* = 0.017) (Fig. 3). There were no significant differences between conditions at T2.

##### DAT

There was a significant effect of treatment on tracking error [*F*(2,54.73) = 5.52, *p* = 0.007], which was increased at T1 in the THC/CBD condition when compared with both the placebo (*p* < 0.001) and THC (*p* = 0.042) conditions (Fig. 3). There were no significant effects on response time or number of items correct and no differences between treatments at T2.

##### PASAT

In the PASAT there was a significant effect of treatment on both number of correct trials [*F*(2,52.52) = 6.30, *p* = 0.004] and response time [*F*(2,52.93) = 11.09, *p* < 0.001] (Fig. 3). Fewer correct trials (*p* < 0.001) and increased response time (*p <* 0.001) were observed at T1 in the THC/CBD condition compared with placebo. The THC condition significantly increased response time (*p* < 0.001), but not differences in number of correct trials at T1 compared with placebo (Fig. 3). There were no significant differences between conditions at T2.

#### Plasma cannabinoid concentrations

**Fig. 4:**
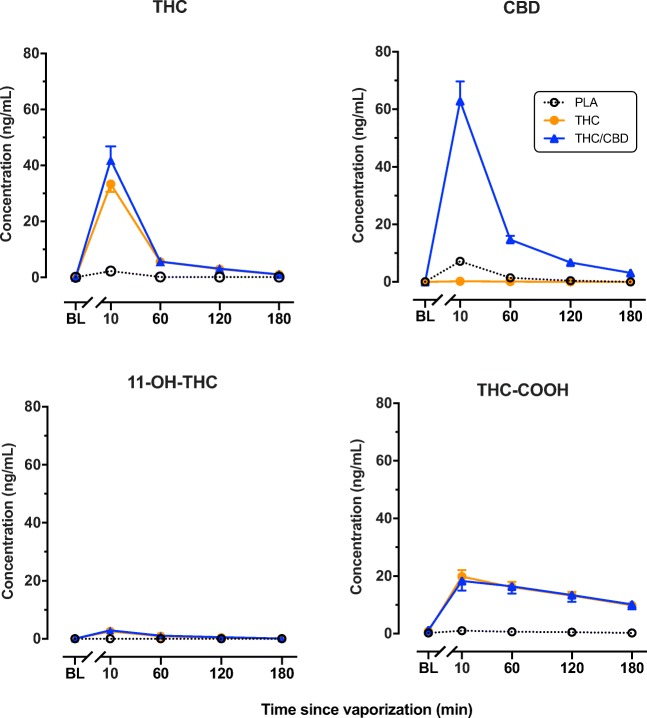
Mean (SEM) plasma concentrations (ng/mL) of THC, CBD, 11-OH-THC, and THC-COOH after vaporization of placebo, THC-dominant, and THC/CBD-equivalent cannabis

Plasma concentrations of THC, its secondary metabolite (11-OH-THC), terminal metabolite (THC-COOH), and CBD, are shown in Fig. 4. Analysis revealed a significant main effect of treatment on plasma THC [*F*(2,96.94) = 83.35, *p* < 0.001], 11-OH-THC [*F*(2,93.86) = 31.37, *p* < 0.001], THC-COOH [*F*(2,136.64) = 93.35, *p* < 0.001], and CBD [*F*(2,107.87) = 136.61, *p* < 0.001]. The main effect of time and the treatment by time interaction was also highly significant in all three models. When compared with the THC condition, the THC/CBD condition displayed increased peak plasma concentrations (*C*_max_) by an additional 8.60 ng/mL (*p* < 0.001). Mean 11-OH-THC *C*_max_ was slightly higher in the THC/CBD condition and THC-COOH *C*_max_ marginally lower, although these differences were not significant. The area under the curve (AUC_0- > 3 h_) for THC was higher in the THC/CBD condition (51.37 ng/mL × h) than in the THC condition (42.51 ng/mL × h), although this difference did not reach significance (*p* = 0.064). 11-OH-THC AUC_0- > 3 h_ was 3.29 (THC) and 4.00 (THC/CBD) ng/mL × h. THC-COOH AUC_0- > 3 h_ was 49.75 (THC) and (THC/CBD) 49.30 ng/mL × h. Mean *T*_max_ was calculated as 0.17 h for all analytes.

## Discussion

Overall, the results of the current study indicate that THC/CBD-equivalent and THC-dominant cannabis produce similar subjective effects and impairment. Vaporization of either chemovar significantly impaired driving during a car-following task and reduced confidence in driving up to 4 h later. Cognitive function was also impaired; however, these effects were more transient and had largely dissipated at the later time point. Pharmacokinetic data showed that peak plasma concentrations of THC appeared higher when CBD was co-administered.

The finding that CBD in cannabis does not greatly alter the subjective effects of THC is consistent with the findings of several previous studies (Hindocha et al. 2015; Morgan et al. 2010a; Roser et al. 2008; Juckel et al. 2007). Both active cannabis types significantly increased ratings of subjective drug effects (e.g., “stoned,” “strength of drug effect”) relative to placebo. These effects were maximal at 15 min and declined steadily thereafter. Participants also expressed markedly reduced confidence in their driving ability immediately after vaporization and even 240 min later. There were only subtle differences between the effects of the two active drug conditions in ratings of “Sedated” or “Anxious,” with THC/CBD equivalent cannabis appearing to cause slightly greater sedation at 240 min and slightly less anxiety at 60 min compared with THC-dominant cannabis.

Both active cannabis types significantly increased SDLP (i.e., lane weaving) during the car-following task at 30 min and 210 min after vaporizing. Although SDLP may not directly predict crash risk, it is a highly sensitive index of THC and alcohol-impaired driving (Veldstra et al. 2015; Bosker et al. 2012; Downey et al. 2013; Hartman et al. 2015; Irwin et al. 2017) and is a widely used proxy for driver safety (Verster and Roth 2011). At the 30-min time point, both the THC and THC/CBD treatments increased SDLP by approximately 4 cm, comparable to the effects of 20 mg dronabinol (Bosker et al. 2012), 19 mg smoked THC (Lenné et al. 2010), and 200-μg/kg smoked THC (equivalent to 15 mg THC for a 75 kg person) (Ramaekers et al. 2000). Participants in both active treatment conditions also tended to leave a larger and more variable gap between them and the lead vehicle, which may indicate an attempt to compensate for perceived impairment (Hartman et al. 2016).

In the secondary driving task, however, neither lateral (SDLP) nor longitudinal (mean speed, SDSP) control measures were affected by cannabis. While the car-following task required participants to constantly monitor and adapt to the speed changes of a lead vehicle while driving in steady highway traffic, the secondary driving task was relatively simple. Although there were some hazards requiring driver interaction (e.g., roadworks), the drive was monotonous and involved long stretches of single-lane rural roads with low traffic density. It is possible that this task underestimated the complexities of real-world driving, although such situations are far from uncommon when driving in rural areas. A recent study similarly found that 13 mg THC (100 mg cannabis containing 12.9% THC) administered via vaporization did not affect simple driving-related task performance despite reducing confidence in driving ability and performance on complex cognitive tasks (Ogourtsova et al. 2018). The impairing effects of THC are known to increase with task complexity (Hartman and Huestis 2013) and therefore may only be evident under demanding driving conditions.

Cannabis administration produced acute cognitive impairment in the DSST, PASAT, and DAT tasks employed in the current study. This agrees with results of a very recent study (Spindle et al. 2018) in which vaporized cannabis (10 mg THC) produced moderate but significant impairment on outcomes in these same tasks. The modest impairment in the DSST involved a significant decrease in correct responses in the THC treatment condition. In the DAT, response time to peripheral stimuli was largely unaffected by cannabis, while tracking error—a measure sensitive to both THC (Verster and Roth 2011; Ramaekers et al. 2009) and alcohol (Jongen et al. 2016)—was increased considerably in the THC/CBD condition relative to the other two conditions. The PASAT was most sensitive to cannabis effects, with processing speed significantly impaired in both the THC and THC/CBD conditions, and number of correct trials significantly decreased in the THC/CBD condition. Collectively, these results suggest that the use of THC/CBD-equivalent cannabis does not prevent the cognitive impairment seen with THC-dominant cannabis and may, under some circumstances, cause greater impairment.

Curiously, at 210 min following vaporization of either THC or THC/CBD, participants still showed impaired lateral vehicular control (i.e., increased SDLP) and reported reduced confidence in driving ability despite an apparent resolution of cognitive impairment and a dissipation of subjective drug effects (e.g. “stoned”). It is thus possible that these cognitive tests (DSST, DAT, PASAT) are only sensitive to the impairing effects of cannabis when the magnitude of effect is large and unambiguous (i.e., during acute intoxication). This may be because these tests predominantly assessed controlled and conscious processes (e.g., response time, attention) that are more accessible to compensatory mechanisms than road tracking (lateral control), which is a highly automated and learned skill that is particularly vulnerable to internal disruptions (e.g., CNS drug effects) (Robbe 1998). It is interesting to note that participants could accurately assess their driving impairment even in the absence of salient subjective drug effects, and that THC-induced driving impairment may persist well beyond the period of acute intoxication. This observation may have implications for the advice given to medicinal cannabis patients around driving safety.

Analysis of plasma cannabinoid concentrations showed that peak THC levels were significantly higher in the THC/CBD condition than in the THC condition. CBD inhibits certain forms of drug metabolism (Stout and Cimino 2014) including those involving the CYP3A4, CYP2C9, and CYP2C19 isoforms involved in THC metabolism (Yamaori et al. 2011). It is conceivable that such inhibition could lead to increased plasma THC concentrations. Two previous studies found no evidence of a pharmacokinetic interaction between THC and CBD with buccal or oral administration in humans (Karschner et al. 2011; Nadulski et al. 2005); however, the maximum CBD concentrations obtained in these studies were an order of magnitude lower than those obtained here with vaporized cannabis. It should also be acknowledged, as an alternative explanation of these results, that THC/CBD equivalent vapor may have a different sensory quality to THC-dominant vapor and that this might lead to subtle differences in dose titration and self-administration, and hence higher plasma THC concentrations with THC/CBD equivalent cannabis. As inhalation remains the most widely used method of both recreational and medicinal cannabis administration, these preliminary findings warrant further investigation and verification.

### Strengths and limitations

Major strengths of this study include a rigorous double-blind, placebo-controlled, within-subjects, and crossover design; the use of the Mighty Medic vaporizer, an approved medical device for cannabis administration in both Canada and the EU; assessment of driving and cognitive function at various timepoints following vaporization; and repeated sampling of blood.

Limitations include the absence of a CBD-only condition: this was omitted because vaporization of CBD alone is uncommon in the real world and because acute administration of CBD in previous human laboratory studies has not produced notable drug effects that are suggestive of intoxication or impairment (Haney et al. 2016; Martin-Santos et al. 2012; Winton-Brown et al. 2011; Borgwardt et al. 2008; Babalonis et al. 2017; Dalton et al. 1976; Hollister 1973). It is also acknowledged that the study used a THC dose sufficient to produce robust subjective and behavioral effects in infrequent cannabis users, but that regular cannabis users may use considerably higher doses than used here. Future studies should therefore consider using multiple THC doses and higher THC and CBD ratios (e.g., 1:10) than the 1:1 ratio that we examined here. Another limitation of this study is the relatively small sample size. The window for participant recruitment was limited by expiration of the study drug and regulatory process in Australia which made further study drug importation difficult. Nonetheless, a range of highly significant results were obtained.

This study was also limited to healthy volunteers who were only occasional cannabis users. Cannabis use history (and therefore tolerance) strongly affects individual responses to THC, with occasional users being significantly more susceptible to impairment than regular users (Bosker et al. 2012; Ramaekers et al. 2009; Desrosiers et al. 2015). Regular cannabis users (i.e., daily or near daily) may therefore be less impaired in the experimental paradigms used here. We also note that while there are obvious advantages to using a driving simulator (i.e., greater experimental control and minimal risk), the complexities of real-world driving may not be replicated entirely. A carefully controlled on-road driving study would therefore be useful in verifying the results obtained here. Further research is also needed to validate the cognitive tasks used here among others as predictors of real-world driving performance.

Finally, the fact that all 14 participants correctly identified the placebo session suggests an issue with placebo cannabis preparations that is difficult to resolve, particularly when it is administered alongside active cannabis in a within-subjects crossover design. Of course, blinding is always a challenge in psychopharmacological research when an active treatment has distinctive and salient psychoactive effects. Placebo cannabis that retains some of the aroma and taste of active cannabis may still be preferable relative to alternatives such as inert herbal mixtures.

## Conclusion

In conclusion, this study indicates that vaporized cannabis with equivalent concentrations of THC and CBD causes similar impairment of driving and cognition to THC-dominant cannabis, and does not produced substantially different subjective effects. In fact, the presence of CBD may increase plasma concentrations of THC, and subtly increase some measures of cognitive and driving impairment. These results have significant implications for individuals using medicinal and recreational cannabis containing both THC and CBD.

## Electronic supplementary material

Figure S1 (PDF 27.4 kb)
