# Supplementary figures and images for: Cannabidiol (CBD) content in vaporized cannabis does not prevent tetrahydrocannabinol (THC)-induced impairment of driving and cognition
Source: Psychopharmacology (Berl). 2019 May 1;236(9):2713–24. doi: 10.1007/s00213-019-05246-8 (PMC6695367; doi:10.1007/s00213-019-05246-8)

## Adelaide Driving Self-Efficacy Scale

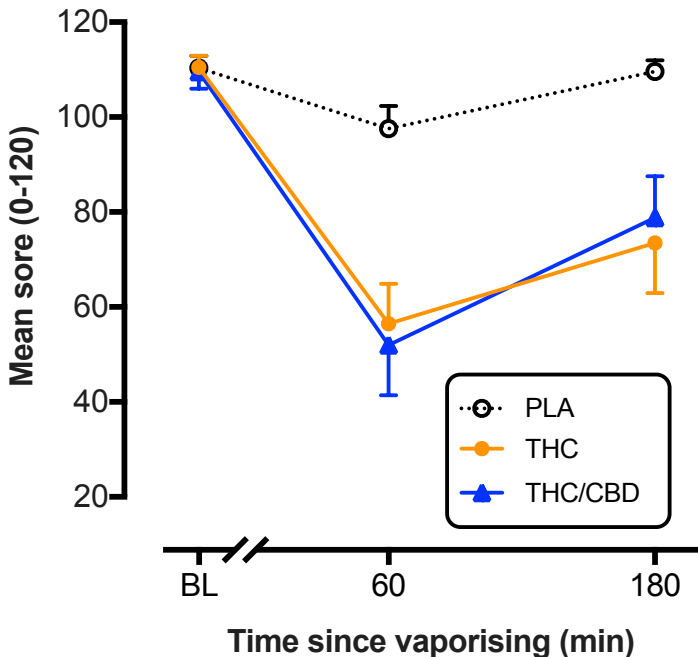

Supplement: Supplementary file 1 — (PDF 27.4 kb) [file 213_2019_5246_MOESM1_ESM.pdf]
